# Audiology practice in assessing and managing tinnitus: a cross-sectional study

**DOI:** 10.3389/fneur.2025.1666022

**Published:** 2025-10-01

**Authors:** Rania Alkahtani, Aryam Alshamardel, Asma Alzakari, Rahaf Alshaya, Sarah Aldawsari, Haya Aldawsari, Haya Frhan, Reem Elbeltagy

**Affiliations:** Department of Health Communication Sciences, College of Health and Rehabilitation Sciences, Princess Nourah Bint Abdulrahman University, Riyadh, Saudi Arabia

**Keywords:** tinnitus, assessment, management, Saudi Arabia, audiology practice

## Abstract

**Objectives:**

Tinnitus may negatively impact quality of life, emphasizing the importance of effective management to support patients’ well-being. This study aimed to gain insights into the current practices among audiologists in Saudi Arabia in assessing and managing tinnitus patients and to identify areas for improvement.

**Methods:**

A cross-sectional study surveyed 96 audiologists using an electronic questionnaire comprising 30 items and 5 demographic questions. The questionnaire covered appointment structure, tinnitus assessment and management, outcome measures, determinants of successful management, clinical skills, resource availability, and satisfaction with services.

**Results:**

Only 14.6% of workplaces had specialized tinnitus clinics, and appointment durations were generally short. Of the audiologists, 32.3% reported practicing multidisciplinary care, while group therapy was not used, and family involvement was acknowledged by less than half. Audiological tests were widely used for assessment, but psychoacoustic measures and validated questionnaires were uncommon. Management primarily relied on hearing aids and counseling, with limited use of psychological approaches such as cognitive behavioral therapy. Audiologists reported moderate satisfaction with their effectiveness in managing tinnitus (mean = 3.25 ± 0.98). Higher satisfaction was associated with working in specialized clinics, multidisciplinary teams, same-day assessment and treatment, and more trained audiologists available for counseling (all *p* < 0.05). However, none of these factors remained significant in regression analysis.

**Conclusion:**

Tinnitus practice in Saudi Arabia revealed notable discrepancies in approaches taken by audiologists, primarily due to limited training and resources. Establishing evidence-based guidelines, expanding professional training, and improving resource allocation are needed to enhance the quality and consistency of tinnitus care, ultimately improving patient outcomes.

## 1 Introduction

Tinnitus is the perception of sound in the absence of an external source (1). It is not considered a disease in itself but rather a symptom of various underlying conditions. The underlying causes of tinnitus are often difficult to pinpoint. It is thought to result from cochlear injuries that lead to peripheral deafferentation, prompting adaptive changes in the central nervous system. It is frequently associated with auditory disorders such as Ménière’s disease, noise-induced hearing loss, and presbycusis (1). Recent studies also suggest that both auditory and non-auditory factors may influence tinnitus, indicating its multifactorial in nature and. For example, cardiovascular risk indicators, including dyslipidemia and hyperglycemia, have been associated with tinnitus occurrences (2). Additionally, hematological parameters such as hemoglobin and platelet counts have been correlated with tinnitus severity, suggesting the involvement of vascular and inflammatory pathways in its pathophysiology (3).

Globally, tinnitus affects around 749 million adults, with 120 million experiencing severe symptoms (4). It can markedly impair QoL, with certain personality traits such as neuroticism, anxiety-proneness, and heightened stress sensitivity exacerbating its perception as a distressing symptom, particularly in those with comorbid illness (5, 6). Chronic tinnitus is associated with reduced QoL through sleep disturbance, cognitive and emotional difficulties, and impaired concentration, often leading to frustration, irritability, and chronic stress. These challenges can interfere with daily functioning, work productivity, and social relationships, and in severe cases may be associated with suicidal ideation (5, 7). Given these wide-ranging consequences, effective assessment and management are essential.

Tinnitus evaluation typically involves a medical history and clinical examination to identify treatable causes (8, 9). Recommended assessments include ear, nose, and throat (ENT) examination, audiological evaluation, and the use of validated questionnaires to assess tinnitus impact (9).

Management approaches are varied. Conservative strategies, such as improving sleep, managing stress, and reducing caffeine, are often advised to reduce symptoms and enhance QoL (8, 10). Evidence-based interventions include behavioral therapy, sound therapy, and medication, while surgery is rarely used and reserved for specific underlying conditions such as Ménière’s disease or tumors (11). Cognitive behavior therapy (CBT) is one of the interventions that has consistently shown effectiveness in improving QoL for tinnitus patients (8, 12). Other options include tinnitus retraining therapy (TRT), melatonin, antidepressants, and cognitive training to address sleep, mood, and cognitive symptoms (8, 13).

The prevalence of tinnitus among adults in Saudi Arabia is 37.6%, with 23% experiencing a mild handicap as measured by the Tinnitus Handicap Inventory (THI). Unfortunately, nearly 60% of individuals with tinnitus did not seek medical support, with reported barriers including the belief that no treatment was available (66.7%), perceiving the condition as tolerable (22.5%), or not knowing which specialty to consult (10.8%) (14). An earlier study conducted in Saudi Arabia in 2011 found that 43% of participants experienced bilateral tinnitus, and 76% had some degree of hearing loss (15).

The high prevalence of tinnitus in Saudi Arabia, combined with misconceptions about its management and the limited research on clinical practices, underscores the need for further investigation. These reported barriers highlight the importance of improving patient awareness, correcting misconceptions about available treatments, and strengthening referral pathways. This study, therefore, aimed to examine current practices among audiologists in assessing and managing tinnitus patients, to identify areas for improvement, and to guide future tinnitus care in the country.

## 2 Methods

### 2.1 Participants and recruitment

A cross-sectional study was conducted over 6 months, from October 2023 to March 2024. Participants were audiologists practicing in Saudi Arabia, recruited through professional networks, including colleagues in hospitals and audiology professional groups. A reminder notification was sent after 7 weeks to enhance participation and response rates.

### 2.2 Materials

The study employed a survey adapted from Hoare et al. (16), with additional items developed to capture demographic information. The questionnaire comprises 30 items along with 5 additional demographic questions. These items addressed clinic structure, patient characteristics, audiologist background, tinnitus assessment, and management (see Supplementary material 1).

### 2.3 Statistical analysis

Statistical analysis was performed using IBM SPSS version 29. Data were analyzed using both descriptive and inferential statistics. Descriptive data were summarized as frequencies and percentages. Responses to the item assessing participants’ satisfaction with the effectiveness of the services provided were recorded on a 5-point Likert scale (1 = highly dissatisfied to 5 = highly satisfied) and treated as a continuous variable. Mean and standard deviation were calculated to describe overall effectiveness scores. Group differences in effectiveness were examined using independent-sample *t*-tests for dichotomous variables (e.g., gender, presence of a specialized clinic, multidisciplinary approach) and one-way ANOVA for variables with three or more categories (e.g., age group, number of patients seen per week, consultation duration). When ANOVA indicated significant differences, *post hoc* comparisons were conducted using Tukey’s test. To identify independent predictors of effectiveness, a multiple linear regression model was fitted, including specialized clinic availability, multidisciplinary approach, same-appointment care, and number of trained audiologists as explanatory variables. Statistical significance was set at *p* < 0.05.

### 2.4 Ethical consideration

Ethical approval was obtained from the Institutional Review Board (IRB) of Princess Nourah bint Abdulrahman University (IRB number: 23–0658). Participation was voluntary, and electronic informed consent was obtained from all participants at the start of the survey.

## 3 Results

A total of 96 audiologists were included in the study. Their demographic information is presented in Table 1. The questionnaire focused on audiologists’ reported practices and did not collect patient-level clinical details such as comorbid hearing loss, vertigo, or age of tinnitus onset. Thus, the findings reflect professional practices rather than specific patient characteristics. The majority of the audiologists (64.6%) reported seeing 1–5 individuals with tinnitus per week, while 21.9% see 6–10 patients and 13.5% see more than 10 patients weekly. Regarding patients’ emotional status at first consultation, audiologists reported that nearly half (47.9%) perceived their patients as distressed, while 36.5% perceived them as neutral and 15.6% as positive.

**Table 1:** Demographics of the audiologists included in the study (*n* = 96).

Factor	*N*	%
Gender		
Female	70	72.9
Male	26	27.1
Age (years)		
21–29	54	56.3
30–39	32	33.3
40–49	7	7.3
50–59	3	3.1
Region of residency		
Central region	61	63.5
Eastern region	14	14.6
Northern region	5	5.2
Southern region	7	7.3
Western region	9	9.4
Nationality		
Saudi	88	91.7
Non-Saudi	8	8.3
Place of work		
Private hospital/clinic	48	50
Public hospital	48	50

### 3.1 Appointment structure

The majority of audiologists (85.4%) reported that their workplace lacked a specialized tinnitus clinic. Referrals most commonly came from ENT specialists (92.7%), while smaller proportions came from GPs (20.8%), self-referrals (33.3%), or other specialists such as neurologists or psychologists (9.4%). Consultation times were typically 15–30 min (87.4%), with only 12.5% of audiologists reporting sessions of 60 min.

Combined assessment and treatment were offered in the same appointment by 25% of audiologists, sometimes by 52.1%, and never by 22.9%. Group therapy was rarely provided (1%). Family involvement in care was also limited: 14.6% included families during assessment, 20.8% during management, and 7.3% during evaluation, while 42.7% restricted family involvement to pediatric cases.

A multidisciplinary approach was reported by 32.3% of audiologists. In this context, a multidisciplinary approach refers to collaboration between audiologists and other health professionals, most frequently ENT physicians (74.2%), followed by neurologists (41.9%), dentists (19.4%), psychologists (16.1%), and psychiatrists (9.7%).

### 3.2 Tinnitus assessment

**Figure 1:**
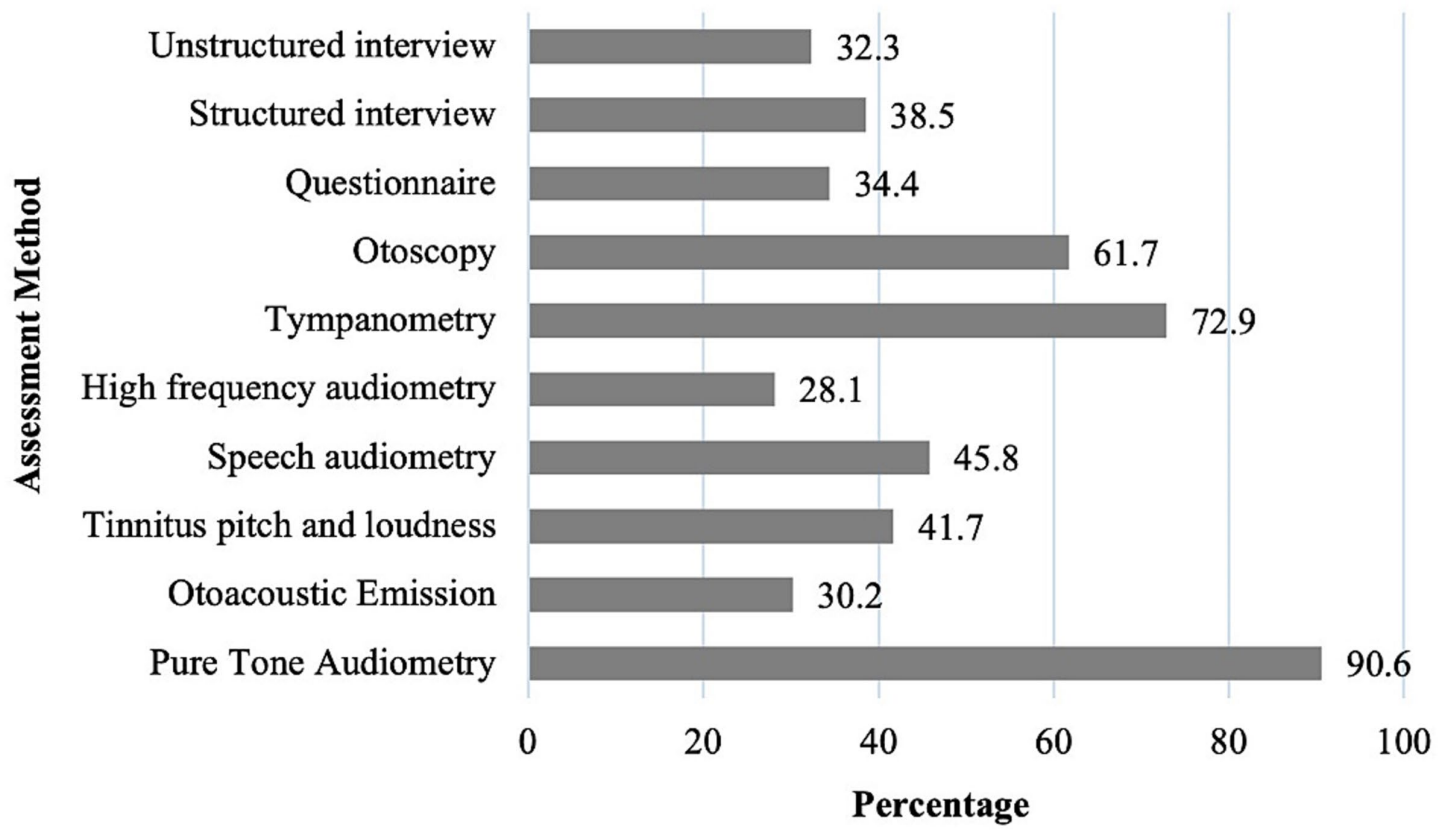
Utilization of different tinnitus assessment methods by audiologists.

Audiologists reported that in their clinical practice, they most frequently use pure-tone audiometry (92.7%), tympanometry (83.3%), and speech audiometry (72.9%) in tinnitus assessment (Figure 1). Fewer reported using tinnitus pitch/loudness matching (30.2%) or high-frequency audiometry (21.9%). Validated tinnitus questionnaires were employed by 34.4%, but only 4.2% specified the THI, and none used validated questionnaires for psychological comorbidities such as anxiety or depression. These findings indicate that traditional audiological measures remain the predominant tools in clinical practice, while psychoacoustic and psychosocial assessments are less commonly integrated into routine evaluation.

Of the total audiologists, 34.7% reported that their departments had standardized assessment protocols, while 24.2% reported none, and 41.1% were uncertain. Most audiologists supported at least partial standardization (90.6%).

### 3.3 Tinnitus management

In the management of tinnitus, audiologists reported using various approaches. The most common management strategies were hearing aids (88.5%) and directive counseling (80.2%). Sound generators (37.5%), TRT (22.9%), stress management (28.1%), CBT (16.7%), and other psychological support (14.6%) were less frequently used. External referral to psychologists or other specialists was available in 40.6% of workplaces. Most audiologists (78.1%) reported applying different criteria for hearing aid fitting in tinnitus patients.

### 3.4 Outcome assessment

**Figure 2:**
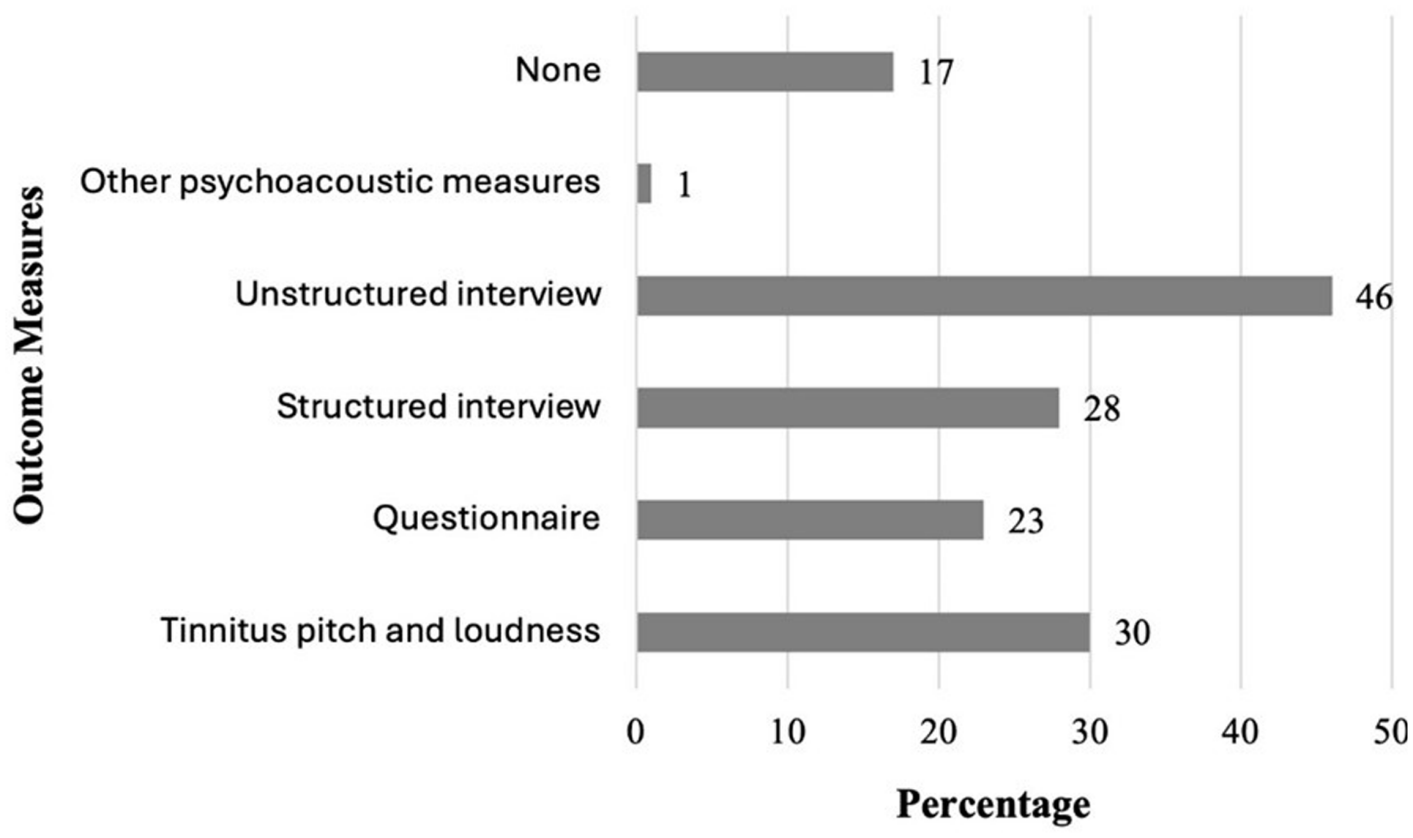
Outcome measures used by audiologists in tinnitus management.

Regarding treatment outcome measures, audiologists reported that in their clinical practice, they most frequently use unstructured interviews with patients (45.8%), followed by structured interviews (28.1%) and questionnaires (22.9%) (Figure 2). 30.2% of audiologists employed objective outcome measures like pitch and loudness matching, while 16.7% did not use any formal outcome measure. Only 5.2% of audiologists who used questionnaires specified the THI. These results suggest that outcome evaluation is often informal and variable across clinics, with limited reliance on standardized or validated measures. Departmental standardization of outcome assessment was uncommon, with only 15.8% reporting standardized procedures, 25.3% reporting none, and 58.9% uncertain.

### 3.5 Clinical skills in tinnitus care, resource availability, and service satisfaction

Training in tinnitus care was limited: 46.3% of audiologists reported no trained colleagues in their workplace, 21.1% reported more than two trained colleagues, 21.1% reported only one, and 11.5% reported two. Most (62%) had not received tinnitus training themselves; those who did mainly attended a comprehensive course (22.9%) or CBT training (12.5%) (Table 2). Just over half (56.2%) had attended a tinnitus seminar in the past 5 years. A majority (55.2%) believed additional training in psychological approaches was necessary, and 44.8% saw it as beneficial depending on need.

**Table 2:** Type of tinnitus training received by audiologists (*n* = 96).

Tinnitus training	*N*	%
A comprehensive course on tinnitus	22	22.9
CBT in audiology practice	12	12.5
1–2 online lectures	2	2.1
None	60	62.5
Total	96	100

Resource availability was also limited: only 19.8% reported having adequate resources, while nearly half (47.9%) did not, and 32.3% were uncertain. Satisfaction with services was mixed: 43.8% rated them effective, 35.4% neutral, and 20.8% dissatisfied.

Audiologists’ self-reported satisfaction with their effectiveness in helping patients manage tinnitus averaged 3.25 ± 0.98 (median = 3.0, range = 1–5), indicating moderate satisfaction overall. Group comparisons (Table 3), conducted using independent-sample t-tests and one-way ANOVA, showed significantly higher satisfaction among audiologists working in specialized clinics (*t* = 2.90, *p* = 0.005), within multidisciplinary teams (*t* = 3.07, *p* = 0.003), offering same-appointment care (*F* = 4.37, *p* = 0.015), and with more trained colleagues available (*F* = 4.86, *p* = 0.004). No significant differences were observed for demographic variables. A multiple linear regression model was conducted with audiologists’ satisfaction with the effectiveness of tinnitus services as the dependent variable. The model, which included practice-related factors (availability of a specialized tinnitus clinic, multidisciplinary team involvement, same-day assessment and treatment, and number of audiologists trained in counseling), explained 22% of the variance (*R*^2^ = 0.217, *p* = 0.002). However, none of the predictors remained statistically significant when considered simultaneously (Table 4), indicating that their effects are likely overlapping and interrelated within well-resourced clinical settings.

**Table 3:** Group comparisons of audiologists’ satisfaction with the effectiveness of tinnitus services.

Factor	Test*	*p*-value
Gender	*t* = 0.82	0.417
Age	*F* = 0.87	0.458
Region	*F* = 2.36	0.060
Workplace	*t* = −1.04	0.302
Number of patients per week	*F* = 0.49	0.617
The availability of specialized tinnitus clinic	*t* = 2.90	0.005**
Multidisciplinary team involvement	*t* = 3.07	0.003**
Same-day assessment and treatment	*F* = 4.37	0.015**
Family involvement	*F* = 2.27	0.086
Type of sessions (individual vs. group)	*F* = 0.96	0.387
Consultation duration	*F* = 0.23	0.798
Number of audiologists trained in counseling	*F* = 4.86	0.004**

*Independent-sample *t*-tests were used for dichotomous variables, while one-way ANOVA was used for variables with three or more categories.

**Statistically significant.

**Table 4:** Multiple linear regression predicts audiologists’ satisfaction with the effectiveness of tinnitus services.

Predictor		*B* (SE)	*t*	*p*-value	95% CI*
Intercept		4.62 (0.64)	7.22	<0.001	3.35, 5.90
The availability of specialized tinnitus clinic		−0.36 (0.30)	−1.21	0.231	−0.94, 0.23
Multidisciplinary team involvement		−0.40 (0.22)	−1.84	0.069	−0.84, 0.03
Same-day assessment and treatment		0.03 (0.11)	0.26	0.797	−0.19, 0.25
Number of audiologists trained in counseling (ref = 1)	2	0.49 (0.35)	1.40	0.166	−0.21, 1.19
	>2	0.11 (0.30)	0.36	0.723	−0.48, 0.69
	None	−0.36 (0.25)	−1.46	0.147	−0.85, 0.13

*CI, confidence interval.

## 4 Discussion

This study provides new insight into audiologists’ current practices in tinnitus care in Saudi Arabia and highlights several important gaps. The main findings revealed that most audiologists underutilized psychoacoustic tests and validated questionnaires, instead relying on standard audiological measures. Management strategies centered mainly on hearing aids and counseling, with limited uptake of evidence-based psychological approaches such as CBT and TRT. Outcome evaluation was inconsistent across clinics, often relying on unstructured interviews rather than standardized tools. Satisfaction with effectiveness was moderate, with group comparisons showing significantly higher satisfaction among audiologists working in specialized clinics, within multidisciplinary teams, offering same-appointment care, and having more trained colleagues. However, regression analysis demonstrated that these factors were interdependent, suggesting that comprehensive, well-resourced clinical environments, rather than single elements in isolation, are most likely to enhance tinnitus care.

One strength of this study is that it is the first to provide national-level data on tinnitus practice in Saudi Arabia, moving beyond prevalence studies to examine how audiologists assess, manage, and evaluate outcomes. When compared with existing literature from other countries, several contrasts become evident. In terms of appointment structure, only 14.6% of audiologists reported having specialized tinnitus clinics within their workplaces, compared with Northern and Southern Europe, where these are more common (17). Appointment times were typically shorter in Saudi Arabia, with half of audiologists offering 30 min, one-quarter 15 min, and only one-quarter 60 min. By contrast, in the UK, average appointments last 60–75 min, while in Northern Europe, they typically range from 30 to 60 min (16, 17). Shorter consultation durations may compromise the comprehensiveness of assessment and limit opportunities for counseling, despite evidence that nearly half of individuals with tinnitus experience significant distress and require psychological support. Referral pathways in this study were broadly appropriate, with patients directed by GPs, ENTs, neurologists, psychologists, or self-referral, consistent with international practices. Increasing community awareness of self-referral options could help reduce delays in accessing services, particularly given that many individuals with tinnitus in Saudi Arabia do not seek medical help (14).

The limited uptake of multidisciplinary care, group therapy, and family involvement also contrasts with evidence showing that these approaches improve adherence, provide psychosocial support, and enhance QoL (9, 18–23). In this study, only 32.3% of audiologists in this study reported participating in multidisciplinary teams, and none reported any involvement in group therapy. Greater expansion of collaborative, patient-centered models of care could therefore strengthen service provision.

Assessment practices showed strong reliance on audiological measures such as pure tone and high-frequency audiometry, speech audiometry, tympanometry, and otoacoustic emissions, consistent with best practice (9). However, psychoacoustic measures (pitch and loudness) and validated questionnaires were underutilized, despite recommendations from international guidelines (9, 24). Only 41.7% of audiologists reported using psychoacoustic measures during assessment, and 25% during follow-up. Although these rates are relatively low, they are still higher than those reported in the UK (17 and 4%, respectively) (16). Recent findings demonstrate that THI scores correlate strongly with tinnitus loudness but not pitch (25), which emphasizes the value of integrating psychoacoustic measurements alongside self-reports. Validated questionnaires such as the THI were also seldom employed (25% at assessment and 23% at follow-up), and no standardized tools were used to assess anxiety or depression. Instead, most audiologists relied on unstructured interviews, which hindered comparability across clinics. These findings highlight an urgent need to increase the uptake of validated measures for both tinnitus severity and psychological comorbidities.

International guidelines for tinnitus practice, such as the Clinical Practice Guideline for Tinnitus developed by Tunkel et al. (24) and the Multidisciplinary European Guideline for Tinnitus developed by Cima et al. (9), recommend incorporating standardized questionnaires and psychoacoustic measures into clinical pathways. However, the lack of validated Arabic-language tinnitus tools presents a barrier in Saudi Arabia. Developing culturally and linguistically appropriate measures, as well as raising awareness of existing Arabic-language instruments (26–28), would allow for more accurate assessment and tailored interventions. Outcome evaluation in this study was highly variable, with 45.8% relying on unstructured interviews, 28.1% on structured interviews, 22.9% on questionnaires, 30.2% on objective measures, and 16.7% reporting no formal evaluation. This heterogeneity illustrates the importance of standardized outcome assessment protocols in clinical practice.

In terms of management, hearing aids and directive counseling were the most commonly used strategies, while sound generators, CBT, and TRT were underutilized. This differs from European practice, where CBT and TRT are more widely implemented (16, 17). CBT is strongly recommended for managing tinnitus-related distress (12, 21, 29, 30), and TRT combines counseling with sound therapy to facilitate habituation (13, 31). The limited application of these therapies in Saudi Arabia likely reflects gaps in professional training. Without adequate preparation in psychological approaches, audiologists are unable to provide comprehensive, interdisciplinary care. Variation in assessment, management, and outcome practices further highlight the absence of national protocols. Implementing standardized guidelines would reduce variability and improve consistency across services (24, 32).

The statistical findings also carry important implications. Audiologists’ mean satisfaction score (3.25 on a five-point scale) indicates only moderate confidence in their effectiveness. Group comparisons showed that higher satisfaction was significantly associated with organizational resources such as specialized clinics, multidisciplinary care, integrated appointments, and trained colleagues. This suggests that structural and organizational support has a major influence on clinicians’ perceived effectiveness. However, the regression model showed that none of these factors remained significant when analyzed simultaneously, suggesting that their effects overlap. In practice, we need comprehensive service environments that combine specialized clinics, collaborative care, integrated appointments, and adequate counseling support to achieve meaningful improvements. This finding aligns with international evidence highlighting the benefits of multidisciplinary and well-resourced care models (9, 19, 20, 32). By contrast, audiologists in the UK and USA report higher satisfaction (16, 33), likely due to greater availability of training and resources.

The study has limitations. The sample size was relatively small, which may reduce the generalizability of the findings. The demographic distribution was unbalanced, with overrepresentation of younger audiologists and those practicing in the central region. The reliance on self-reported data introduces recall and social desirability bias, and structured survey responses may not capture the full range of clinical practices. Finally, the survey was designed to capture audiologists’ practices rather than patient-level clinical data, so details on comorbid conditions or age of tinnitus onset were not collected. These limitations suggest that findings should be interpreted with caution.

Overall, the findings reveal systemic challenges: limited specialized services, inadequate use of standardized assessment tools, and restricted access to evidence-based psychological approaches. Importantly, no national clinical protocols currently exist, emphasizing the pressing need for evidence-based guidelines to standardize care and reduce variability across clinics. At the same time, opportunities for improvement were identified: investing in training, expanding workforce capacity, and strengthening interprofessional collaboration could support the development of a more consistent and effective model of tinnitus care.

## 5 Conclusion

Tinnitus practice in Saudi Arabia shows notable gaps, particularly in training, standardized protocols, and resource availability. Targeted actions, such as implementing evidence-based guidelines, expanding specialized training, and improving resources, are essential to strengthen care. Addressing these gaps will enhance service delivery, patient satisfaction, and overall quality of life for individuals with tinnitus.

## Data Availability

The raw data supporting the conclusions of this article will be made available by the authors, without undue reservation.
