# Supplementary material for: Audiology practice in assessing and managing tinnitus: a cross-sectional study
Source: Front Neurol. 2025 Oct 1;16:1666022. doi: 10.3389/fneur.2025.1666022 (PMC12520938; doi:10.3389/fneur.2025.1666022)
Supplement: Supplementary file 1 [file Data_Sheet_1.PDF]

Questionnaire

Do you agree to participate in the study by filling up this short questionnaire?

- [ ] Yes
- [ ] No

Age

- [ ] 21-29
- [ ] 30-39
- [ ] 40-49
- [ ] 50-59
- [ ] 60 or older

Gender

- [ ] Male
- [ ] Female

Region of residency

- [ ] Central region
- [ ] Northern region
- [ ] Southern region
- [ ] Eastern region
- [ ] Western region

Nationality

- [ ] Saudi
- [ ] Non-Saudi

Place of work

- [ ] Public hospital
- [ ] Private hospital/clinic

Is there a specialized clinic for tinnitus in your workplace?

- [ ] Yes
- [ ] No

What referral pathway for tinnitus patients is used in your workplace? (select all that apply)

- [ ] GP
- [ ] ENT
- [ ] Direct
- [ ] Other (please specify): ____________________

Is there a multidisciplinary approach to treat the tinnitus patient in your workplace?

- [ ] Yes
- [ ] No

If yes, which professionals are included? (select all that apply)

- [ ] ENT
- [ ] Audiologist

- [ ] Psychologist
- [ ] Psychiatrist
- [ ] Physiotherapist
- [ ] Neurologist
- [ ] Dentist
- [ ] Other (please specify):

Do you offer a tinnitus assessment and treatment in the same appointment?

- [ ] Yes
- [ ] No
- [ ] Someties

Do you actively encourage family involvement?

- [ ] In the assessment stages
- [ ] In the treatment stages
- [ ] In the treatment outcome stages
- [ ] Family is not involved unless if the patient is a child

What sort of clinical appointments do you routinely offer?

- [ ] One-to-one with the individual
- [ ] Individual and a family member
- [ ] Group sessions (more than 2)

Do you consider tinnitus to be…?

- [ ] Symptom

- [ ] Disease
- [ ] Audiological problem
- [ ] Psychological problem
- [ ] Other (please specify):

How many tinnitus patients do you see in one week?

- [ ] 1-5
- [ ] 6-10
- [ ] More than 10

Do you see predominantly…?

- [ ] Chronic tinnitus (more than 3 months)
- [ ] Acute tinnitus (less than 3 months)
- [ ] Both

How much time can you allocate to an individual tinnitus patient in one consultation?

- [ ] 15 min
- [ ] 30 min
- [ ] 60 min
- [ ] More than 60 min

What is on average the emotional status of your patient at the moment of first consultation?

- [ ] Very positive
- [ ] Somewhat positive

- [ ] Neutral
- [ ] Distressed
- [ ] Very distressed

How many individuals in your clinic are trained specifically to provide counseling or other psychological support to people with tinnitus?

- [ ] 1
- [ ] 2
- [ ] More than 2
- [ ] None

What tinnitus training have you received?

- [ ] None
- [ ] Tinnitus comprehensive Course
- [ ] CBT in audiological practice
- [ ] Other (please specify): ____________

What is your view on audiologists undertaking additional training in psychological approaches to tinnitus management?

- [ ] Necessary
- [ ] Possible according to need
- [ ] Not necessary

Did you attend a tinnitus conference/seminar in the last 5 years?

- [ ] Yes
- [ ] No

What diagnostic tools/approach do you use on a tinnitus patient? (select all that apply)

- ☐ Structured interview
- ☐ Unstructured interview
- ☐ Questionnaires
- ☐ Otoscopy
- ☐ Tympanometry
- ☐ Pure tone audiometry
- ☐ High frequency audiometry (12, 16, 20 kHz)
- ☐ Speech audiometry (hearing loss)
- ☐ Tinnitus pitch and loudness
- ☐ OAE
- ☐ Other (please specify):

If you use questionnaires, please specify what questionnaires do you use?

Do all tinnitus specialists in your clinic use the same assessment protocol?

- ☐ Yes
- ☐ No
- ☐ Not sure

In your view, how standardized should the tinnitus assessment procedure be?

- [ ] Completely standardized
- [ ] Fairly standardized
- [ ] Non standardized

What formal treatments are offered at your clinic? (select all that apply)

- [ ] Directive counseling (information and reassurance)
- [ ] Hearing aids
- [ ] Sound generators
- [ ] Tinnitus retraining therapy (or a version of)
- [ ] Stress management
- [ ] Cognitive-behavioural therapy
- [ ] Other form of psychological support
- [ ] Other (please specify):

Do you have the option to refer your tinnitus patients to a clinical psychologist or other specialist outside the clinic who is qualified in providing psychological therapy?

- [ ] Yes
- [ ] No

Do you use different criteria for offering hearing aids to people who do or do not have tinnitus?

- [ ] Yes
- [ ] No

What methods do you use for assessing treatment outcome? (select all that apply)

- [ ] None
- [ ] Structured interview
- [ ] Unstructured interview
- [ ] Questionnaires
- [ ] Objective measures (e.g., tinnitus pitch and loudness)
- [ ] Other (please specify):

If you use questionnaires, please specify what questionnaires do you use?

Is the measurement of treatment outcomes standardized across all tinnitus specialists in your clinic?

- [ ] Not applicable
- [ ] Yes
- [ ] No
- [ ] Not sure

What do you consider to be important factors that determine a successful outcome from treatment?

- [ ] Cure of tinnitus
- [ ] Improve quality of life
- [ ] Other (please specify):

Do you feel that you have access to sufficient resources to provide an effective tinnitus service?

- [ ] Yes
- [ ] No
- [ ] Not sure

In general, are you satisfied with the services provided to patients with tinnitus?

- [ ] Highly Satisfied
- [ ] Satisfied
- [ ] Neither Satisfied nor Dissatisfied
- [ ] Dissatisfied
- [ ] Highly Dissatisfied
